# Fractional Anisotropy of Thalamic Nuclei Is Associated With Verticality Misperception After Extra-Thalamic Stroke

**DOI:** 10.3389/fneur.2019.00697

**Published:** 2019-07-17

**Authors:** Taiza E. G. Santos, Jussara A. O. Baggio, Carlo Rondinoni, Laura Machado, Karina T. Weber, Luiz H. Stefano, Antonio C. Santos, Octavio M. Pontes-Neto, Joao P. Leite, Dylan J. Edwards

**Affiliations:** ^1^Department of Neurosciences and Behavioral Sciences, Ribeirao Preto Medical School, University of São Paulo, São Paulo, Brazil; ^2^Moss Rehabilitation Research Institute, Elkins Park, PA, United States; ^3^School of Medical and Health Sciences, Edith Cowan University, Joondalup, WA, Australia

**Keywords:** verticality perception, diffusion tensor imaging, diaschisis, thalamus, stroke

## Abstract

Verticality misperception after stroke is a frequent neurological deficit that leads to postural imbalance and a higher risk of falls. The posterior thalamic nuclei are described to be involved with verticality perception, but it is unknown if extra-thalamic lesions can have the same effect via diaschisis and degeneration of thalamic nuclei. We investigated the relationship between thalamic fractional anisotropy (FA, a proxy of structural integrity), and verticality perception, in patients after stroke with diverse encephalic extra-thalamic lesions. We included 11 first time post-stroke patients with extra-thalamic primary lesions, and compared their region-based FA to a group of 25 age-matched healthy controls. For the patient sample, correlation and regression analyses evaluated the relationship between thalamic nuclei FA and error of postural vertical (PV) and haptic vertical (HV) in the roll (PV_roll_/HV_roll_) and pitch planes (PV_pitch_/HV_pitch_). Relative to controls, patients showed decreased FA of anterior, ventral anterior, ventral posterior lateral, dorsal, and pulvinar thalamic nuclei, despite the primary lesions being extra-thalamic. We found a significant correlation between HV_roll_, and FA in the anterior and dorsal nuclei, and PV_roll_ with FA in the anterior nucleus. FA in the anterior, ventral anterior, ventral posterior lateral, dorsal and pulvinar nuclei predicted PV, and FA in the ventral anterior, ventral posterior lateral and dorsal nuclei predicted HV. While prior studies indicate that primary lesions of the thalamus can result in verticality misperception, here we present evidence supporting that secondary degeneration of thalamic nuclei via diaschisis can also be associated with verticality misperception after stroke.

## Introduction

Verticality misperception is the incapacity to judge the orientation of the body or environment in relation to *Earth vertical*, within normal limits (1, 2). This frequent neurological deficit leads to postural imbalance exposing patients to a higher risk of falls after stroke (3, 4). Deficits in the important sub-components, postural (PV) and haptic vertical (HV) are associated with postural dysfunction post-stroke even in the absence of clinical signs (3, 4).

The structural biology of verticality perception is incompletely understood; however, studies of voxel-based lesion-behavior mapping and neuronal network-based mathematical modeling (5–7), have shown a clear involvement of posterior nuclei of the thalamus. What remains unclear is whether degeneration of the thalamic nuclei is a common feature when primary lesions are outside thalamus, and whether symptom-lesion association is confined to specific posterior nuclei of the thalamus. We sought to answer these questions using Diffusion Tensor Imaging (DTI), to quantitatively analyze microstructural integrity of thalamic nuclei, and examine diaschisis (8–10). Diaschisis can be defined as selective change in coupling between two nodes of a defined network, involving areas distant from the lesion (8).

DTI studies using fractional anisotropy (FA) can quantify microstructural characteristics of brain tissue, reflecting axonal properties in the white matter and cytoarchitecture in the gray matter (11, 12). The FA of the thalamic nuclei has been investigated in several studies because it is composed of both gray matter and myelinated fibers (9, 13). Moreover, FA maps showing secondary lesions of the thalamus have been correlated with clinical symptoms after encephalic lesions (9, 14). However, until now, no study has investigated if microstructural integrity of the thalamus or other brain regions is correlated with verticality misperception in patients after encephalic lesions. Therefore, we assessed HV, PV, and DTI metrics of a random sample of post-stroke patients with diverse encephalic lesions that spared the thalamus. Here we present a cross-sectional study showing that verticality misperception in post-stroke patients can occur with primary lesions outside the thalamus and is related to fractional anisotropy of thalamic nuclei.

## Methods

We used a cross-sectional observational study design. Sixteen right-handed patients sequentially admitted to the Emergency Unit of our institution diagnosed with first time stroke (excluding; <18 years, thalamic stroke, other neurological conditions, altered level of consciousness, cognitive deficit, receptive aphasia, and severe concomitant systemic illness) enrolled in the study. A control group comprising 25 right-handed healthy subjects matched by age and gender was also investigated.

For the stroke sample, documented clinical features included; handedness (Edinburgh Questionnaire), vascular risk factors (arterial hypertension, heart disease, history of smoking, diabetes, alcoholism, and sleep apnea), Mini-Mental State Exam (MMSE), National Institutes of Health Stroke Scale (NIHSS), modified Rankin Scale (mRS), Barthel Index (BI), Functional Independence Measure (FIM), Ability for Basic Movement Scale (ABMS), Scale for Contraversive Pushing (SCP), and spatial neglect. PV and HV were quantified in the roll (PV_roll_/HV_roll_) and pitch (PV_pitch_/HV_pitch_) planes using a standard protocol previously reported by our group (4) and others (1), with error angle measured using a digital inclinometer accurate to 0.01°. HV and PV tilt error (degrees from vertical) toward or away from the encephalic lesion (positive or negative, respectively), was described as (1) mean of real numbers (+ or −), abbreviated as PVr and HVr, and (2) mean of absolute numbers (independent of sign), abbreviated as PVa and HVa (see Supplementary Methods).

Magnetic Resonance Images (MRI) were acquired with an Achieva 3T X-Series MRI scanner (Philips, Best, The Netherlands) using an 8-channel head coil. Anatomical 3-D T1-weighted images were acquired from each participant using the following parameters: TR = 7 ms; TE = 3.201 ms; flip angle = 8 degrees; matrix = 256 × 256; FOV = 256 mm; voxel size = 1 × 1 × 1 mm. Axial T2 and volumetric FLAIR sequences were conducted to investigate white mater hyperintensities in stroke patients and classified using the total score of the Fazekas scale (sum of periventricular and deep white matter hyperintensities) (15). MRI scans were examined for possible brain edema, which can distort the DTI analysis.

The DTI sequence was used to quantify fractional anisotropy. To improve the accuracy of the eigenvector estimation in each voxel, data was obtained in 32 different directions of diffusion gradients. The volumes had 72 axial images of 2 mm of thickness with a FOV of 256 × 256 mm (matrix of 128 × 128, 2 mm isotropic voxel). Repetition time (TR) was 8,391 ms with 65 ms an echo time (TE). In total, 33 volumes were collected, 32 of which had a diffusion weighting of 1,000 s/mm^2^. Motion correction was applied to the diffusion volumes using the standard scanner software.

The MRI data were analyzed using BrainVoyager QX 2.8 (Brain Innovations, The Netherlands). The locations and boundaries of the lesions were delineated on the individual MRI scans by one neurologist (LHS) and one neuroradiologist (ACS) that were blind to subject verticality estimates. Additional description of the lesions was made regarding the brain areas previously related to verticality misperception including; insula (1, 16–18), parietal cortex (1, 16, 17, 19), superior and middle temporal gyrus (1, 18–20), temporo-parietal junction (21–23), post central gyrus (16, 18), and inferior frontal gyrus (1, 18, 19). The first volume of the DTI acquisition was aligned to the anatomical T1-weighted image through an affine transformation. The T1-weighted image was also nonlinearly registered to the Talairach template using trilinear interpolation for standardization in the 3D space. Talairach space was chosen in this study due to its deterministic nature and because subcortical structures and small ROIs are relatively invariant in shape after imaging normalization techniques (24). The lesion shapes were manually delineated using the corregistered FLAIR images on the background of the T1-weighted structural MRI.

Regions-of-interest from the Talairach template were used to extract values from the following ipsilesional locations: pulvinar, anterior nucleus, dorsal nucleus, mammillary body, medial dorsal nucleus, midline nucleus, ventral lateral nucleus, ventral posterior lateral nucleus, ventral posterior medial nucleus, ventral anterior nucleus (Supplementary Figure 1). In the control group, the right hemisphere was selected to analyze FA, being the dominant hemisphere for verticality perception, and more representative of our stroke sample. We also expressed the thalamic nuclei FA in the affected hemisphere relative to the FA in the unaffected hemisphere using an established lateralization index (25, 26), and repeated the same analysis for control subjects to compare left and right hemispheres. The index is defined as follows:

$$\begin{array}{l} Lateralization\;index\;controls=\;\left(L-R\right)/\left(L+R\right)\\ \;L=left;R=right\\ Lateralization\;index\;patients=\;\left(FA_{u}-FA_{a}\right)/\left(FA_{u}+FA_{a}\right)\\ \;FA_{u}=FA\;of\;unaffected\;hemisphere;\\ \;FA_{a}=FA\;of\;the\;affected\;hemisphere \end{array}$$

### Statistical Analyses

Statistical analyses were performed using SPSS v20.0. All tests were two-sided and considered significant at the 95% level (α = 0.05). Comparison between the stroke and control groups for gender was determined using the Pearson Chi-Square test, and the Mann-Whitney test for age, FA of thalamic nuclei and lateralization index.

To investigate if secondary lesions of the thalamic nuclei are related to verticality misperception, we used the Spearman's rank correlation coefficient. Here we analyzed the correlation between perception of verticality, and FA of the specific thalamic nuclei that showed significantly higher values in controls, relative to post-stroke patients.

The forward stepwise multiple regression considered verticality perception as the dependent variable and FA of the thalamic nuclei of stroke group (that were significantly different from the control group, using the Mann-Whitney test) as independent variables.

## Results

The authors declare that all supporting data are available within the article and its online supplementary files (Supplementary Data 1).

Four patients were excluded due to midline shift and one due to movement artifact that would bias the DTI analyses. The stroke group characteristics were: mean age 64.5 ± 14.4 years; 54.5% females; median NIHSS score 2 (0–3); mean MMSE score 23.4 ± 3.4; median modified Rankin Scale score 3 (1–3); mean BI score 83.5 ± 18.9; mean FIM 105.6 ± 18.5; mean ABMS score of 18.1 ± 3.1; median SCP score 0 (min = 0; max = 0.25); and 72.7% presented with upper limb sensory deficit (item 8, NIHSS). The mean time from stroke to the evaluation was 32.7 ± 11.9 days and the MRI scans were acquired within 20 days of clinical assessment.

Risk factors for stroke in patient sample were as follows: 72.7% arterial hypertension, 27.3% heart disease, 27.3% history of smoking, 18.2% diabetes; 9.1% alcoholism, and 9.1% sleep apnea.

**Figure 1:**
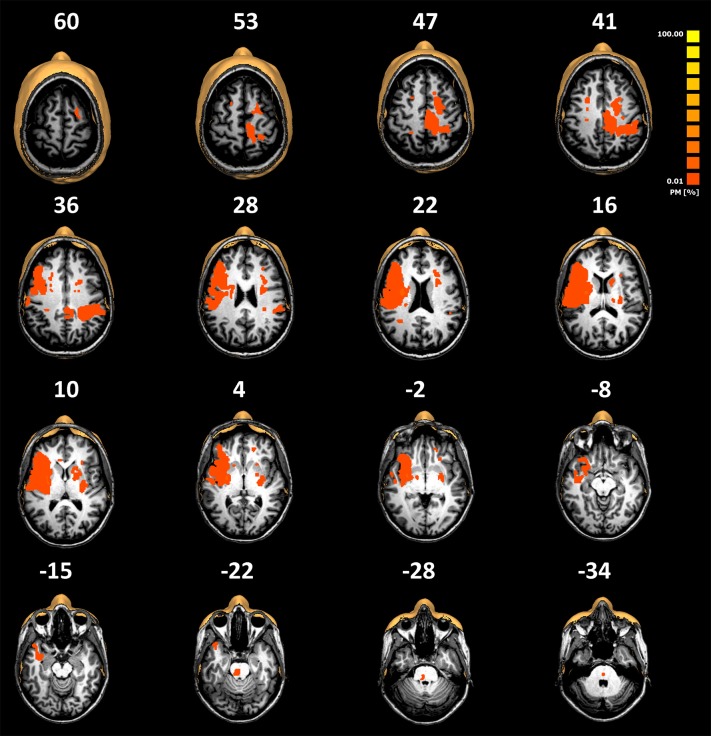
Normalized lesion probability maps from the group of patients overlaid on selected slices of the normalized T1-weighted MRI scans of a representative patient (patient number 5 described in Table 1). Lesions have been manually plotted using the BrainVoyager QX software. The low overlay percentage shows the high variability of the lesion locations observed in our cohort. Note that there are no primary thalamic lesions.

White matter hyperintensities observed in our sample were classified (see Table 1), and as per previous reports in healthy subjects of comparable age, showed the highest proportion in category 1 and a lower proportion in category 3 (27). Lesion probability maps from the group of patients are illustrated in Figure 1.

**Table 1:** Summary of injury characteristics and descriptive values of verticality perception for each post-stroke patient.

**Post-stroke patient #: age range**	**HVa HVr**	**PVa PVr**	**HVa HVr**	**PVa PVr**	**Lesion side**	**Lesion location**	**Lesion location related to verticality misperception**	**Risk factors**	**Fazekas scale**
	**Roll plane**		**Pitch plane**						
P1: 70–75	3.86 0.41	3.35−0.37	7.42 7.42	3.093.09	RH	Posterior internal capsule and corona radiata	–	Hypertension, cardiac disease, smoking	2
P2: 80–85	5.84 −4.24	1.040.80	3.69 −0.75	6.12−4.84	LH	Temporal lobe, corona radiata, putamen and globus pallidus	Temporo-parietal junction, parietal lobe	Sleep apnea	1
P3: 70–75	11 −10.33	1.83−1.50	7.48 7.48	2.13−0.62	RH	Frontal and parietal lobe	Parietal lobe	Hypertension, alcoholism	3
P4: 70–75	5.15 5.15	1.521.52	10.55 10.55	3.643.02	LH/BS	Corona radiata and Pons	–	Hypertension, cardiac disease, diabetes	3
P5: 30–35	3.43 −0.24	4.151.19	4.11 3.57	1.89−0.75	RH	Parietal lobe	Parietal lobe	Cardiac disease	1
P6: 50–55	11.78 −11.78	1.710.24	3.40 −2.31	2.03−1.29	RH	Frontal lobe, caudate nucleus and putamen	Inferior frontal lobe	No	2
P7: 75–80	5.35 5.35	1.5−1.00	6.58 6.15	1.201.20	LH	Frontal lobe	–	Hypertension	2
P8: 60–65	3.37 −2.45	2.31−0.01	3.06 3.06	1.71−1.39	RH	Frontal lobe and corona radiata	–	Hypertension, smoking	1
P9: 50–55	3.99 −1.92	3.822.13	9.18 9.18	2.772.41	RH	Globus pallidus	–	Hypertension	3
P10: 65–70	7.64 −7.42	2.991.57	10.70 0.60	3.15−0.81	RH	Frontal and parietal lobe	Parietal lobe	Hypertension	1
P11: 65–70	1.18 −1.18	1.3−0.29	5.99 2.96	2.47−0.98	BS	Pons	–	Hypertension, diabetes, smoking	2

*Anatomy codes: WM, white matter; GM, gray matter; RH, right hemisphere; LH, left hemisphere; BH, bilateral hemisphere; BS, brain stem; FL, frontal lobe; TL, temporal lobe; PL, parietal lobe; OL, occipital lobe; R, right; L, left*.

The control group was matched by age (mean = 63 ± 9.3 years, *p* = 0.199, *z* = −1.283) and gender (14 female/11 male, *p* = 0.732). Relative to the control group, stroke patients showed significantly reduced FA of the following thalamic nuclei: pulvinar, anterior nucleus, dorsal nucleus, ventral posterior lateral, ventral anterior. The descriptive FA values of each group and comparisons of FA between groups are described in Supplementary Table 1.

Further analyses of the relationship of unaffected hemisphere to affected hemisphere in stroke patients supports our primary finding, with significantly higher FA lateralization index relative to the FA lateralization index in control subjects for the following thalamic nuclei: pulvinar (*p* = 0.032); dorsal nucleus (*p* = 0.007); mammillary (*p* = 0.038), and ventral posterior lateral (*p* = 0.006) (Supplementary Table 2).

The following are median values for verticality perception error; PVr_roll_ 0.24° (range: −1.5° to 2.13°), PVr_pitch_ −0.75° (range: −4.84° to 3.09°), PVa_roll_ 1.83° (range: 1.04° to 4.15°), PVa_pitch_ 2.47 (range: 1.2° to 6.12°), HVr_roll_ −1.92 (range: −11.78° to 5.35°), HVr_pitch_ 3.57° (range: −2.31° to 10.55°), HVa_roll_ 5.15° (range: 1.18° to 11.78°), HVa_pitch_ 6.58° (range: 3.06° to 10.7°).

**Figure 2:**
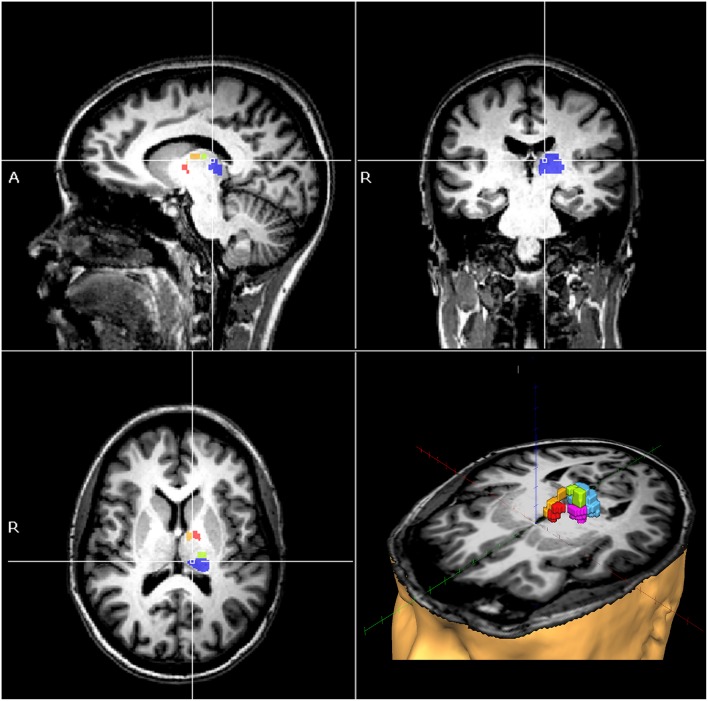
MRI of a single patient as an example, showing the pulvinar (blue), anterior (yellow), ventral anterior (red), dorsal (green), and ventral posterior lateral thalamic nuclei (magenta), where fractional anisotropy values were shown to be independent predictors of verticality perception in post-stroke patients.

We found a significant correlation between PVa_roll_ with FA of the anterior nucleus (*r* = 0.724; *p* = 0.012; Supplementary Figure 2), and between HVa_roll_ with the FA of the anterior nucleus (*r* = −0.615, *p* = 0.044; Supplementary Figure 3), and dorsal nucleus (*r* = −0.624, *p* = 0.040; Supplementary Figure 4) in stroke patients. The results of the additional analyses using the Pearson correlation test between verticality perception and FA of the thalamic nuclei are described in Supplementary Results 1. The regression models showed that FA of the anterior, ventral anterior, dorsal, and ventral posterior lateral nuclei predicted PVa_roll_ (*R*^2^ = 0.962, adjusted *R*^2^ = 0.936, *f* = 37.629, *p* < 0.001); FA of the pulvinar, anterior and ventral anterior nuclei predicted PVr_roll_ (*R*^2^ = 0.656, adjusted *R*^2^ = 0.509, *f* = 4.454, *p* = 0.047); FA of the dorsal, ventral posterior lateral nuclei predicted HVa_roll_ (*R*^2^ = 0.649, adjusted *R*^2^ = 0.561, *f* = 7.402, *p* = 0.015); FA of the ventral anterior and ventral posterior lateral nuclei predicted HVr_pitch_ (*R*^2^ = 0.534, adjusted *R*^2^ = 0.417, *f* = 4.577, *p* = 0.047). The regression models showed no significant relationship between FA of the thalamic nuclei and PV in the pitch plane. Figure 2 shows the thalamic nuclei found to be associated with verticality perception.

## Discussion

Our findings show that verticality misperception can occur from diverse encephalic lesions associated with secondary changes in structural integrity of thalamic white matter, but without primary stroke lesion to the thalamus. We note that our finding of verticality misperception in the current cohort of consecutively admitted acute stroke patients without lateropulsion behavior, is consistent with our prior report of subclinical postural anomaly (4), and raises whether verticality misperception is more prevalent in stroke than currently considered.

In the present study, we showed the association of fractional anisotropy (FA) of thalamic nuclei with two different measures of verticality perception, *postural* and *haptic* vertical. Both of these measures involve multisensory processing and higher order integration, requiring capabilities such as awareness, attention, memory, and three-dimensional perception, and in the case of haptic, motor execution (28–30). Previous evidence indicates that several thalamic nuclei participate in one or more of these capabilities (28, 29, 31, 32). Therefore, in addition to the ventral lateral posterior nuclei of the thalamus historically related to verticality misperception in stroke patients via primary lesion-mapping (5, 6), our data implicate additional thalamic nuclei in the neural network that processes verticality perception.

Here we show with established outcome measures, the additional contribution of the pulvinar, anterior, ventral anterior and dorsal nuclei in verticality perception, providing more insight for the neuroanatomical basis of important sub-systems for the upright body posture. In this context, the anterior and ventral anterior nuclei have been described to participate in spatial navigation and memory (29). The pulvinar is an associative thalamic nucleus topographically organized (33) with strong connections to frontal, prefrontal, and parietal and temporal cortices (31, 32). These areas are described to be part of the neural network related to verticality perception (1, 18), via primary lesion association. The dorsal nucleus has first, and higher order connections with sensory and motor systems, and dense intra-thalamic connectivity (34). Therefore, our argument of thalamic nuclei related to verticality perception found in this study is in agreement with the neurofunctional and neuroanatomical literature.

The negative correlation observed between HV and FA of both the anterior, and the dorsal nuclei, reflect the direct association of decreased thalamic integrity with worse verticality perception. The FA of one thalamic nucleus (anterior nucleus) was positively correlated with one measure of verticality perception (PVa_roll_). This indicates more accurate postural verticality perception, associated with decreased FA. While in contrast to our primary hypothesis, the association of decreased FA in one (or more) node of a neural network, with better neurological performance, has been previously described (35, 36). These findings indicate that future studies should analyze individual nuclei as done here, and consider that observed behavior is the consequence of network-level interaction. Moreover, since diaschisis can occur due to both anatomical and functional disconnection (8), multi-modal investigations that combine structural imaging, functional imaging and verticality perception measures will also be necessary to elucidate the presence of connections between the thalamic nuclei and the primary encephalic lesion associated with verticality misperception.

It should be noted that some stroke patients in our sample scored from 1 to 3 in the Fazekas scale, potentially indicative of microangiopathy. However, hyperintensities in this range can be observed in healthy subjects of comparable age, and thus the contribution to verticality misperception, such as in the present study, requires further investigation.

Here we analyzed PV, HV, and FA as continuous variables, and only included patients with at least one type of verticality misperception. The relationship of verticality misperception with thalamic FA reported here provides preliminary support for the role of thalamic nuclei in such misperception. However, causality requires further exploration, such as studies using a case-controlled design to compare the structural integrity of thalamic nuclei in patients with, and without, verticality misperception.

Furthermore, Wallerian degeneration in the central nervous system observed by FA has been described to decrease monotonously during the first 3 months after stroke, then stabilizes (37, 38). The present preliminary study has acquired the MRI data within the 3 months after stroke onset, which may have input higher variability of FA values. Future studies may acquire MRI data within narrower time or after 3 months of stroke onset to decrease group variability. As well, differences between subjects due to demographic characteristics, lesion location, rehabilitation treatments, and neuroplastic idiosyncrasies are also factors to be considered for the interpretation of the results. Additionally, the external validity of the present findings may be examined in future studies with a larger sample size.

This study provides evidence supporting future investigations of white matter integrity post-stroke, further exploring the association of verticality misperception with primary and secondary lesions of thalamic nuclei.

## Ethics Statement

This study was conducted according to the Helsinki Declaration requirements for human investigation and was approved by the Ethics Committee for Analysis of Research Projects, Hospital das Clínicas de Ribeirao Preto, University of Sao Paulo (HCRP no 3157/2011). All participants provided written informed consent.

## Author Contributions

TS, JB, JL, AS, and DE: study concept and design. TS, JB, KW, LM, LS, CR, JL, and DE: data acquisition and analysis. TS, JB, and DE: statistical analysis. TS, JB, and CR: manuscript writing. LS, LM, KW, AS, OP-N, JL, and DE: critical revision of the manuscript.

### Conflict of Interest Statement

The authors declare that the research was conducted in the absence of any commercial or financial relationships that could be construed as a potential conflict of interest.
