# Supplementary material for: Fractional Anisotropy of Thalamic Nuclei Is Associated With Verticality Misperception After Extra-Thalamic Stroke
Source: Front Neurol. 2019 Jul 17;10:697. doi: 10.3389/fneur.2019.00697 (PMC6650785; doi:10.3389/fneur.2019.00697)
Supplement: Supplementary file 1 [file Data_Sheet_1.docx]

**SUPPLEMENTARY MATERIAL**

**Fractional anisotropy of thalamic nuclei is associated with verticality misperception after extra-thalamic stroke**

Taiza EG Santos, PhD; Jussara AO Baggio, PhD; Carlo Rondinoni, PhD; Laura Machado, PT; Karina T Weber MSc; Luiz H Stefano, MD; Antonio C Santos, PhD; Octavio M Pontes-Neto, PhD; Joao P Leite, PhD; Dylan J Edwards, PhD.

**Supplementary Data 1.** Dataset for results presented

**Supplementary Methods 1.** Clinical features

Patients were classified as having spatial neglect when there was well-defined evidence of typical clinical behaviors such as spontaneous ipsilesional deviation of eyes and head, orienting to the ipsilesional side when addressed from the front or the contralesional side, and ignoring of contralesional located objects or people.

To assess postural vertical (PV), subjects were seated on a chair with mechanically adjustable tilt, and the legs, thighs, and trunk lightly restrained by bands and pads, head stabilized by a neck brace, and feet unsupported. The subjects were randomly tilted to either side away from true vertical between 15° to 40°, at a maximum velocity of 1.5°/s to avoid semicircular canal stimulation. Subjects were then asked to inform the examiner of the direction to return to vertical, and to stop the chair rotation when in the upright position, at which point error angle from true vertical was recorded. To assess haptic vertical (HV), subjects grasped a polyamide bar (4 cm diameter, 40 cm length; patients unaffected hand, healthy subjects right/dominant hand) that allowed rotation about a central axis, and was mounted on the wall, with height adjusted according to subject comfort. The bar was randomly tilted by the examiner in either direction to at least 15° from vertical, and the subject was required to return the bar to their perceived vertical, then error angle from true vertical was recorded. To guarantee understanding of the procedure, all subjects practiced at least six trials of each verticality modality. Ten trials for each modality were performed and recorded. HV and PV tilt error (degrees from Earth vertical) toward or away from the encephalic lesion (positive or negative, respectively), was described as: (1) mean of *real* numbers, abbreviated as HVr and PVr, representing the average of error angles (positive and negative) across individual subject trials, and (2) mean of *absolute* numbers, abbreviated as PVa and HVa, representing the average of error angles (considering error angle as an absolute value) across individual subject trials. PV and HV data are described in degrees (°). In the roll plane, for stroke patients a positive sign indicated an ipsilesional error and a negative sign a contralesional error. In the pitch plane, a positive sign indicated a forward error and a negative sign a backward error.

**Supplementary** **Results 1.** Pearson correlation test

The Pearson correlation test showed a significant correlation between HVr_roll_ with the FA of the pulvinar (r=-0.604, p=0.050), and between PVa_roll_ with FA of the anterior nucleus (r=0.910; p<0.001).

**Supplementary** **Table 1.** Mean and standard error of fractional anisotropy for each ROI in both groups.

| **Thalamic ROI** | **Post-stroke patients** | | **Control group** | |  |
| --- | --- | --- | --- | --- | --- |
|  | **Mean** | **SE** | **Mean** | **SE** | **p-value** |
| **Pulvinar** | 0.240 | 0.020 | 0.284 | 0.006 | **0.022** |
| **Anterior nucleus** | 0.182 | 0.024 | 0.257 | 0.009 | **0.006** |
| **Dorsal nucleus** | 0.266 | 0.019 | 0.311 | 0.007 | **0.029** |
| Mammillary body | 0.282 | 0.017 | 0.273 | 0.007 | 0.959 |
| Medial dorsal nucleus | 0.236 | 0.019 | 0.267 | 0.005 | 0.236 |
| Midline nucleus | 0.200 | 0.016 | 0.233 | 0.011 | 0.096 |
| Ventral lateral nucleus | 0.304 | 0.016 | 0.324 | 0.006 | 0.154 |
| Ventral posterior medial nucleus | 0.304 | 0.017 | 0.312 | 0.009 | 0.744 |
| **Ventral anterior nucleus** | 0.273 | 0.021 | 0.346 | 0.009 | **0.002** |
| **Ventral posterior lateral nucleus** | 0.371 | 0.026 | 0.421 | 0.015 | **0.048** |

Results of the Mann-Whitney Test. ROI, region of interest; SE, standard error.

**Supplementary** **Table 2.** Mean and standard error of fractional anisotropy lateralization index for each ROI in both groups.

| **Thalamic ROI** | **Post-stroke patients** | | **Control group** | |  |
| --- | --- | --- | --- | --- | --- |
|  | **Mean** | **SE** | **Mean** | **SE** | **p-value** |
| **Pulvinar** | -0.033 | 0.039 | -0.058 | 0.142 | **0.032** |
| Anterior nucleus | -0.038 | 0.041 | -0.066 | 0.018 | 0.363 |
| **Dorsal nucleus** | 0.050 | 0.025 | -0.037 | 0.014 | **0.007** |
| **Mammillary body** | -0.038 | 0.015 | 0.007 | 0.013 | **0.038** |
| Medial dorsal nucleus | 0.062 | 0.039 | -0.009 | 0.011 | 0.103 |
| Midline nucleus | -0.063 | 0.053 | -0.070 | 0.035 | 0.693 |
| Ventral lateral nucleus | 0.036 | 0.018 | -0.005 | 0.012 | 0.077 |
| Ventral posterior medial nucleus | -0.044 | 0.021 | -0.049 | 0.013 | 0.823 |
| Ventral anterior nucleus | -0.015 | 0.044 | -0.018 | 0.014 | 0.481 |
| **Ventral posterior lateral nucleus** | 0.006 | 0.032 | -0.098 | 0.019 | **0.006** |

Results of the Mann-Whitney Test. ROI, region of interest; SE, standard error.

**
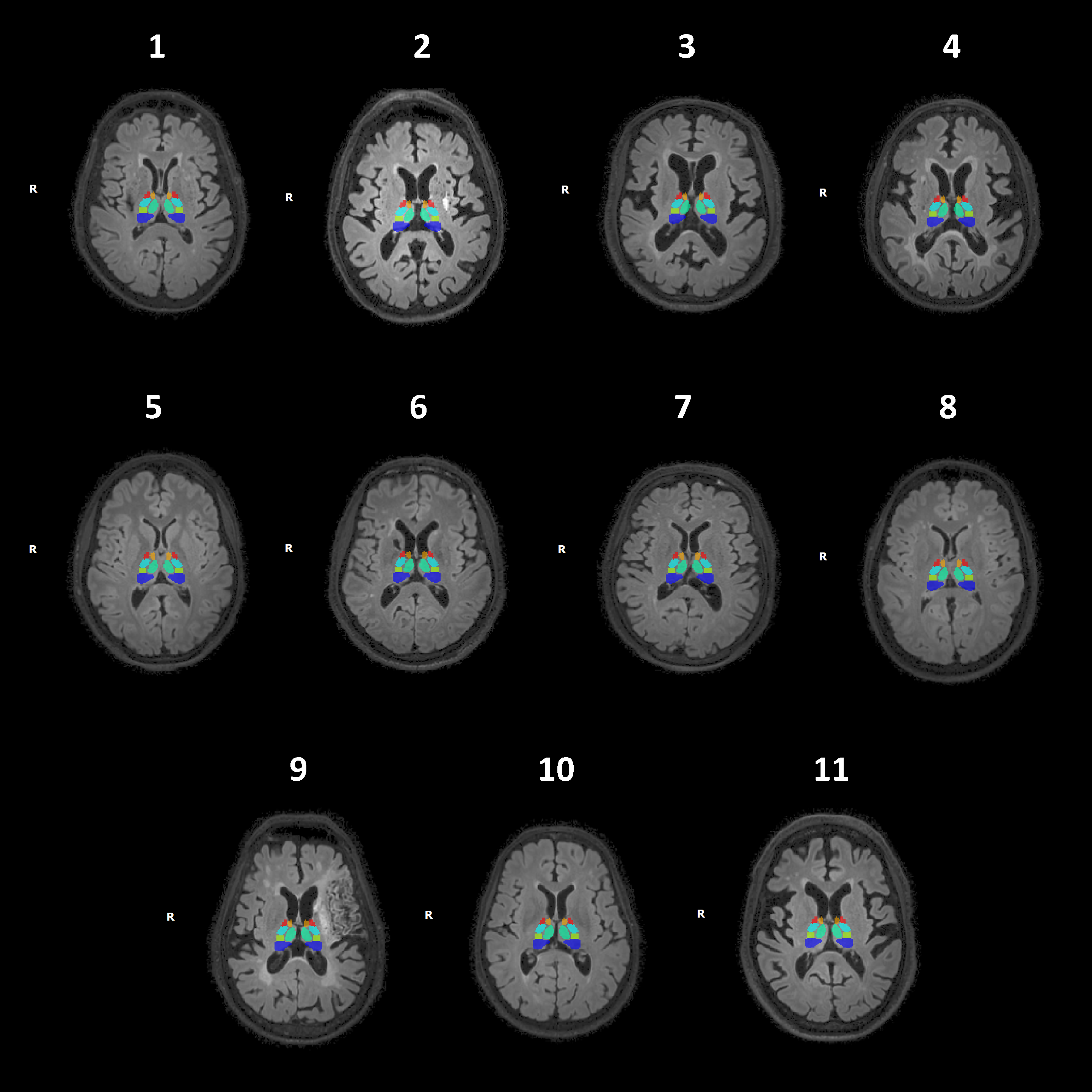
Supplementary** **Figure 1.** Thalamic nuclei ROIs overlaid on the registered FLAIR images of each patient. Note that the modalities of this image overlay and the actual FA analysis are different both in nature and in resolution. FLAIR images are reconstructed with 1mm voxels while DTI images, upon which FA values are calculated, have a voxel resolution of 2mm. Thalamic nuclei are coded in colors: pulvinar (dark blue), dorsal (green), medial dorsal (teal), ventral lateral (light blue), anterior (orange), ventral anterior (red).

**Supplementary Figure 2**. Correlation between Postural Vertical (PV) absolute numbers in the roll plane and the Fractional anisotropy (FA) of the anterior nucleus.


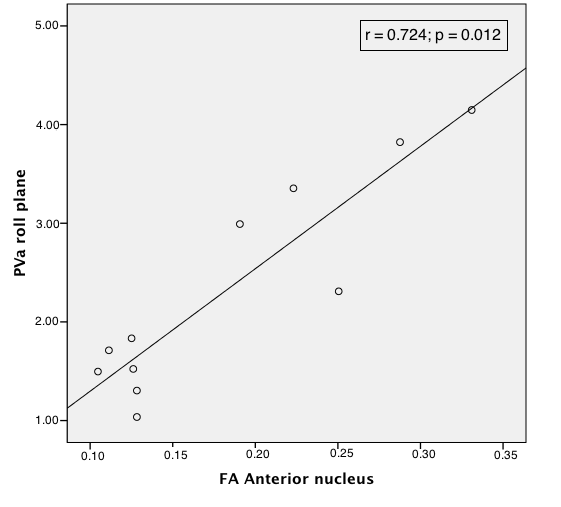


FA: Fractional anisotropy; PVa: Postural Vertical absolute numbers

**Supplementary** **Figure 3.** Correlation between Haptic Vertical (HV) absolute numbers in the roll plane and the Fractional anisotropy (FA) of the anterior nucleus.


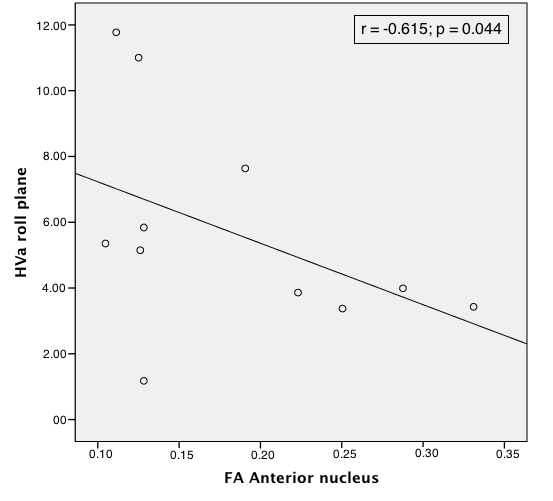


FA: Fractional anisotropy; HVa: Haptic Vertical absolute numbers

**Supplementary** **Figure 4.** Correlation between Haptic Vertical (HV) absolute numbers in the roll plane and Fractional anisotropy (FA) of the dorsal nucleus.


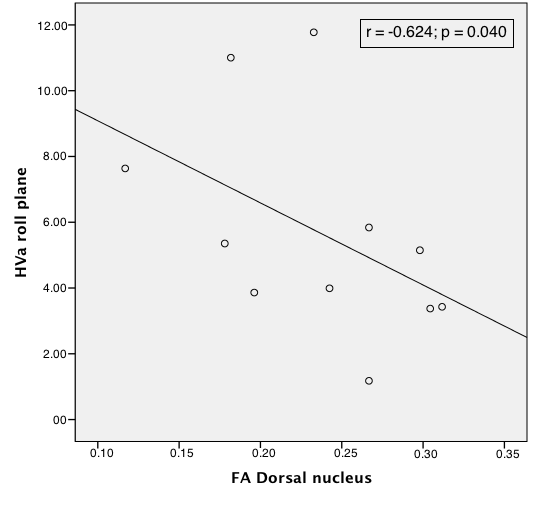


FA: Fractional anisotropy; HVa: Haptic Vertical absolute numbers
